# An Optimal Pollution Control Model for Environmental Protection Cooperation between Developing and Developed Countries

**DOI:** 10.3390/ijerph17113868

**Published:** 2020-05-29

**Authors:** Liyuan Liu, Jing Zhu, Yibin Zhang, Xiding Chen

**Affiliations:** 1 School of Mathematics, Physics and Statistics, Shanghai University of Engineering Science, Shanghai 201620, China; 21180014@sues.edu.cn; 2 School of Business Administration, Southwestern University of Finance and Economics, Chengdu 611130, China; zhuj@swufe.edu.cn; 3 School of Business Administration, Shanghai Lixin University of Accounting and Finance, Shanghai 201209, China; zhangyb@lixin.edu.cn; 4 Department of Finance, Wenzhou Business College, Wenzhou 325035, China

**Keywords:** environment protection, economic growth, greenhouse gas emissions, game theory

## Abstract

With the continuous increase in greenhouse gas emissions in the world and the United States announcing withdrawal from the Paris Agreement, the conflicts between environmental protection and economic growth of developing and developed countries have become increasingly challenging. In this paper, following the principle of “common but differentiated responsibilities” specified in the Kyoto Protocol and the Paris Agreement, we develop an optimal pollution control model based on a dynamic system for both developing and developed countries. We analyze how different perspectives of the developing and developed countries affect their investments in pollution control and how to determine their responsibilities based on the principle of common but differentiated responsibilities. Our aim is to obtain a stable equilibrium mechanism to maximize the social welfare between the developing and developed countries and explore the optimal pollution control and economic growth path. Our results show that it is optimal for the developed countries to help developing countries with pollution control in their initial stage of economic growth. Once the developing countries reach a certain economic development level, they can contribute more to pollution control, while the developed countries can reduce their environmental investment. We show that by following this optimal path, the developing and developed countries can effectively control environment pollution without significant loss of social welfare.

## 1. Introduction

With the increase in greenhouse gas emissions, the conflicts between environmental protection and economic growth have become increasingly challenging for many countries. On one hand, economic development and industrialization have consumed a large amount of resources like energy and water. This has polluted our environment and endangered our sustainable development. To achieve pollution control, various international agreements have been negotiated and reached all over the world. Firstly, the Kyoto Protocol was agreed among developing and developed countries in 1997. As a result, countries have adopted the objective of the United Nations Framework Convention on Climate Change (UNFCCC) to control global warming by reducing greenhouse gas concentrations in the atmosphere to a level that would prevent dangerous anthropogenic interference. Then, partly due to its deficiency and limitations, the Kyoto Protocol was replaced by the Paris Agreement in 2016 [1]. The Paris Agreement provides an opportunity for 196 countries to work together in greenhouse gas emission mitigation and financing within the UNFCCC. However, under the Paris Agreement, several developing countries are unwilling to act promptly, and the developed countries are unwilling to bear the long-term unfair and large responsibilities for environment protection and retreat. For example, China does not plan to fully reduce pollution until 2050. Greece and Hungary pledged to close their coal-fired power plants by 2028 and 2030, respectively [2]. On 1 June 2017, United States President Donald Trump announced that the U.S. would cease all participation in the 2015 Paris Agreement on climate change mitigation. Trump stated that “The Paris accord will undermine (the U.S.) economy”, and “puts (the U.S.) at a permanent disadvantage” [3].

To encourage the wider adoption of the 2015 Paris Agreement, it is clear that further details needed to be negotiated on how the agreement would be implemented transparently and fairly for all. The Katowice Climate Package sets a deadline to complete these negotiations on the implementation guidelines in 2018 at COP24 [4]. The package sets out the essential procedures and mechanisms that will make the Paris Agreement operational. The successful adoption of the well-crafted implementation guidelines is promising to build greater trust and strengthen international cooperation in one of the greatest challenges of our time [5].

To further reduce greenhouse gas emissions and to prevent the mean global temperature from rising by more than 1.5 °C above preindustrial levels, the 2019 UN Climate Action Summit was held at the headquarters of the United Nations in New York City on 23 September 2019 [6]. In the Summit, 60 countries announced to take some steps “to reduce emissions and support the populations most vulnerable to the climate crisis” [7]. The results of the summit were significant, although are believed to be insufficient to prevent the rise of global temperature to less than 1.5 degrees as needed to address the climate crisis. In particular, China did not increase its Paris agreement commitments, India did not pledge to reduce its use of coal, and the U.S. did not even speak at the Summit [8].

To incorporate the UNFCCC, CMP15 (CMP15 means the 15th session of the Conference of the Parties serving as the Meeting of the Parties to the Kyoto Protocol), and CMA2 (CMA2 means the 2nd session of the Conference of the Parties serving as the Meeting of the Parties to the Paris Agreement), the 2019 United Nations Climate Change Conference was held in Spain in December 2019 under the presidency of the Chilean government. Unfortunately, no agreement was reached when concrete climate actions and measures are urgently needed [9]. A prevalent opinion was summarized: “Climate blockers like Brazil and Saudi Arabia, enabled by an irresponsibly weak Chilean leadership, peddled carbon deals and steamrolled scientists and civil society” [10] and the United States, Russia, India, China, Brazil, Saudi Arabia were the main opponents of these measures [11].

Therefore, each country has its own tradeoff between pollution reduction and economic growth. Although the principle of “common but differentiated responsibilities” was specified in the Kyoto Protocol and the Paris Agreement, there is a lack of equilibrium and punishment mechanisms to incentivize the developed and developing countries to invest in environmental protections. These lead to inefficiencies when some International Environmental Agreements (IEA) are implemented in practice.

## 2. Literature Review

The “common but differentiated responsibility” of developed and developing countries to mitigate climate change is the core principle of international climate politics. This principle has evolved from the Kyoto Protocol to the Paris Agreement and the climate governance structure has fundamentally changed [12]. In the mid-2000s, the international community entered into a new period of controversial negotiations of global climate change policies. The Kyoto Protocol adopted the top-down climate governance structure, in which the quantitative national performance standards (the emission levels) are determined by the top management of the Agreement Organization and then the emission levels allocated to each delegate [13].

Acknowledging industrialized countries’ responsibilities for climate change, the international community had exempted developing countries from obligations to cap greenhouse gas emissions under the Kyoto Protocol [14]. In the international negotiations of a deal that followed the Kyoto Protocol, the roles of formerly developing and newly emerging economies such as Brazil, China, India, and South Africa became strongly contested due to their rapid economic growth and consequent increase in greenhouse gas emissions [15]. Thus, between 2004 and 2015, while the international community was negotiating an agreement to enhance the Kyoto Protocol, the international debate focused on how industrialized countries and newly emerging economies should equitably share the burden of climate change mitigation [16]. In contrast, the Paris Agreement adopted a hybrid climate governance structure, in which top-down and bottom-up structures were used, but focused on the latter [17]. Here, the bottom-up structure means that each delegate determined its own quantitative national performance standard (i.e., the emission levels) and submits to the Agreement Organization.

The Kyoto Protocol includes International Emissions Trading and the Clean Development Mechanism and Joint Implementation, which incentivize countries, especially developing ones, to decrease carbon emission [18]. To facilitate more countries to participate, although giving up the dichotomy of “developed countries” and “developing countries”, the Paris Agreement still upholds “the principle of common but differentiated responsibilities” [19]. The Paris Agreement emphasizes more on “built-in flexibility” to distinguish between developed and developing countries’ capacities [20]. The Agreement also developed a Capacity-Building Initiative for Transparency to assist developing countries in building the necessary institutions and processes. Under the Paris Agreement, each country must determine, plan, and regularly report on its contributions to global warming mitigation [21]. However, under the Paris Agreement, should developed or developing countries take the main responsibilities for environmental protection in line with economic development? It is imperative to determine their responsibilities based on the principle of common but differentiated responsibilities.

In most models of the international environmental problems above, the inefficiencies appear when their results are put into practice. Researchers have attempted to address these issues from different aspects. For example, Zhang et al. [22] and Conconi [23] explored whether environmental regulations should be carried out locally or centrally. They found that local control is better when there is sufficient synergy among pollutants. Hoel [24] extended this into transboundary pollution and international trade and examined the trade and environmental policies in two large countries that are linked by trade flows and transboundary pollution. It was found that there is a Nash equilibrium in which each country chooses a policy resulting in lower welfare than would otherwise be possible given the countries’ emission levels. The results showed there is a prisoner’s dilemma game among these countries.

Bhagwati [25] suggested that all countries establish a global warming fund to legitimize the common responsibility of emission reduction. Eyckmans and Finus [26] suggested that a cooperative mechanism be set up to resolve the transboundary pollution problem. Funfgelt and Schulze [27] studied the formation and interaction of environmental pollution policies between two small countries and found that the greater the degree of environmental pollution and the more interested the governments are, the higher environmental taxes would be. He and Zhang [28] evaluated the coordination of industrial-economic development based on anthropogenic carbon emissions. They found that the economic and environmental development appeared coordinated among cities at the same prefecture level, but coordination degrees among different prefecture-level cities vary significantly. Gong et al. [29] proposed an improved three-stage data envelopment analysis model to measure the environmental-economic efficiency of air pollution control for 30 province-level regions of China during 2012 to 2016. Their model considered the factors of capital, labor, and total energy consumption, and variables of gross domestic product and waste gas emissions. In most models above, the lack of international cooperation causes no inefficiency within each country. For the global environment, however, there is no supranational regulating agency which can dictate nations which can enforce environmental standard/regulations to affect the behavior of convening countries. Hence, improvement can only be achieved by voluntary cooperation involving many countries with diverse interests. However, given the target emission levels, there is no effective cooperative mechanism between the developing and developed countries.

A second type of inefficiency is that given the total emission amount in the non-cooperative outcome, it is allocated across countries in an inefficient manner. An externality is an important instance of market failure which produces a deviation from the first-best (Pareto optimal) solution. The problem is that market prices may not necessarily reflect the true social costs or benefits. In such cases, regulatory institutions and instruments are needed. For the global environment, however, improvements can only be achieved by voluntary cooperation involving many countries with very diverse interests. Jørgensen et al. [30] gave a significant literature review on the design of International Environmental Agreements (IEA) and the problem of economic growth under noncooperative game and cooperative games. In a noncooperative game, because participation in an IEA is inherently voluntary, it is necessary to search for mechanisms that lead to the largest possible and stable coalition of countries that adhere to IEA. In a cooperative game, all countries promise to coordinate emissions and find allocations of the joint burden which achieve intertemporal the stability of the agreement and the fairness for the best environmental and economical outcomes.

Noncooperative dynamic games applied to IEAs are still in their infancy, with only several studies with the participation decision as an element of the game. Rubio and Casino [31] analyzed a game where the decision regarding joining an agreement or not is made once and for all, and signatories and non-signatories select their emission strategies in an infinite horizon differential game. Their numerical results show that the stable size of the agreement is two. When requiring a minimum number of signatories for the agreement to be in force, a stable agreement has precisely the same size as in the minimum clause. In Rubio and Casino [31], the dynamics of the pollution stock affected the choice of emission strategies, but not the IEA membership.

In a cooperative dynamic game approach to IEA, the starting point is the grand coalition. A main objective is to identify mechanisms that guarantee the intertemporal stability or sustainability of the grand coalition. Missfeldt [32] discussed the problem of free riding on pollution reduction agreements. By deviating from its part of the cooperative agreement, an agent or group of agents can be better off. Jørgensen and Zaccour [33] studied a differential game where industrial activities of an upstream country pollute a downstream one. Their results suggest a side payment rule that allocates over time the surplus gained if players coordinate their downstream pollution control policies. The rule allocates to each country at each instant of time its share of instantaneous dividend of cooperation based on the egalitarian principle, plus a term that depends on the imputed value of the deviation between the cooperative and noncooperative trajectories of the stocks of pollution and abatement capital. They further showed that side payments always flow from the polluted (vulnerable) country to the nonvulnerable one. Thus, it is more efficient for the vulnerable country to “purchase” a reduction of emissions upstream rather than suffering from its damage. The conditions under which cooperative outcomes are time consistent were also discussed.

The study on the interaction between economic growth and environmental problems was not fully initiated until the 1970s, when exhaustible natural resources and pollution were incorporated in neoclassical growth models. Jørgensen et al. [30] presented a typical differential game model of economic growth and transboundary pollution. They considered two countries producing a single homogenous good that can be consumed or invested in productive capital. The government in each country has a positive and constant rate of time preference and chooses its time paths for consumption and emissions in order to maximize the flow of discounted utility over an infinite horizon. Cabo and Martín-Herrán [34] presented a North–South trade differential game to study how transfers from North to South affect capital growth and biodiversity conservation of the ecosystems in the South. Biodiversity losses affect both economies. North agrees to transfer income to South to diminish biodiversity losses. When North controls the transfer rate and the savings rate is constant, an increase in the latter improves biodiversity conservation. Martin and Meyer [35] presented recent projections from the dynamic Multi-Region Input-Output (MRIO) simulation model. They provided the detailed material footprint and climate policy assessment, indicating a global agreement on a hybrid policy to facilitate climate change mitigation and a sustainable use of natural resources without deteriorating economic performance.

The existing studies mainly focus on the tradeoffs between economic growth and environmental problems using approaches of noncooperative and cooperative games. The literature on dynamic games in pollution control and environmental economics in general has often adopted a modeling strategy of simplifying and downscaling to a limited number of links and players. This has allowed researchers to construct a body of knowledge on the economics of pollution control in a strategic and dynamic setting. The cooperative game approach to the formation and sustainability over time of an IEA has been successfully extended to a dynamic setting. Therefore, research is needed to better understand the formation of large and dynamically stable IEAs, taking into consideration factors such as heterogeneity of players, uncertainty in climate systems, and the principle of common but differentiated responsibilities. To avoid these gaps, in this paper, we propose an optimal pollution control model based on a dynamic system for both developing and developed countries. We are able to identify a stable equilibrium mechanism to maximize the social welfare between the developing and developed countries and the optimal pollution control and economic growth path.

The paper makes the following contributions to the literature. First, it builds one of the first optimal pollution control models based on a dynamic system. Second, it identifies the optimal pollution control path to explore the international cooperation for pollution control and hence sustainable economic growth between developing and developed countries. Third, our results prove that it is optimal for developed countries to assist in pollution control for developing countries in their early stage of economic development. In contrast, in the later stage of economic development, developing countries need to contribute more, while developing countries can contribute less. By following this optimal path, countries can control environment pollution without significant loss of social welfare.

The rest of this paper is structured as follows. Section 2 introduces the theoretic model. Section 3 first identifies the four regions of environmental investment of developing and developed countries and then identifies the boundary among four regions. Section 4 discusses the equilibrium of the optimal pollution control model as well as the economic growth path of the developing countries. Section 5 concludes the paper and proposes several future research directions.

## 3. The Theoretical Model

In our theoretical model, for analytical tractability, we assumed a world economy that is composed of a developing and a developed country denoted by superscripts of 1 and 2 (i=1, 2), respectively. There is transboundary pollution and international trade to flow between the two countries. Let $C_{i}$(i=1, 2) denote the variable consumption and $E_{i}$(i=1, 2) denote the environmental investments, which can be investments in upgrading to energy-efficient production technology or technology transfer. $j_{i}\;$(i=1, 2) is the coefficient (i.e., development levels) of environment technology. $K_{i}=K_{i}\left(t\right)$ (i=1, 2) is the stock of capital at period t. When t=0, the initiate stock of capital is $K_{0,i}$. Thus, ${\dot{K}}_{i}=dK_{i}/dt$ indicates the rate of increase of this capital stock and represent the level of economic growth in both counties.

For the developed country (i=2), let $X_{2}$ denote the fixed component of the environmental investment, which may include fixed investment from government purchase and spending and capital accumulation from trade of goods. $E_{2}$ represents the variable component of its environmental investment and $Y_{2}$ denotes the disposable income in the developed country. For simplicity, we assumed no population growth. The GDP per capita in each country is measured by its average income (or the output) based on its population of the year. Thus, GDP per capita in each country can be used to describe its economic growth. In the developed country, we have $\;GDP_{2}=Y_{2}+X_{2}$. Here, we have $Y_{2}=C_{2}+E_{2}$. The social welfare, which is expressed by a utility function, depends on the consumption and the quality of environment. The utility $U_{X2}$ from $X_{2}$ in the developed country will be dropped in later discussions since its consumption level is smooth in the long run [36].

The objective function is the sum of utility discounted value for the two countries as follows:

(1) $$U\left(Z,\;t\right)=\int_{0}^{\infty }\left[U_{1}\left(C_{1},\;Z\right)+U_{X2}+U_{2}\left(C_{2},\;Z\right)\right]e^{-\theta t}dt.$$

In the dynamic systems, $U_{X2}$ is a fixed parameter and θ indicates time discount rate. Z denotes the stock of pollution in in both countries, and depends on the level of environmental investments in both countries $E_{i}$ and the consumption $C_{i}$, which are the decision variables for both countries, i.e., $Z=Z\left(K_{i},E_{i}\;\right)$. The utility of each country $U_{i}\left(C_{i},\;Z\right)\;\left(i=1,\;2\right)$ is dependent on the consumption $C_{i}\;$ and the stock of pollution Z. Therefore, the objective function may be rewritten as following and each country can maximize their utility to determine their optimal decision:

(2) $$\mathrm{Max}\;U\left(Z,\;t\right)=\int_{0}^{\infty }\left[U_{1}\left(C_{1},\;Z\right)+U_{2}\left(C_{2},\;Z\right)\right]e^{-\theta t}dt,$$

(3) $$K_{1}=F_{1}\left(K_{1}\right)-C_{1}-E_{1}-\delta_{1}K_{1},$$

(4) $$Z=G_{1}\left(K_{1}\right)-j_{1}E_{1}-j_{2}E_{2},$$

(5) $$Y_{2}=C_{2}+E_{2},\;K_{1}\left(0\right)=K_{0,i},$$

where $\delta_{1}$ denotes the depreciation rate in the developing country and $\;F_{1}=F_{1}\left(K_{1}\right)$ indicates the total output of developing country. $\delta_{1}K_{1}$ indicates a rate of stock of capital, which depends on international industry chain configuration, advanced environment technology in the developed country, and lower economic development rate [30]. We assumed that the emission volume is in stable state in the developed country in its production and consumption and the intensity of environment pollution depends on the stock of capital in the developing country [30]. Hence, the environment pollution intensity of the whole system depends on the stock of capital in the developing country ($K_{1}$), the environmental investment ($K_{i}\geq 0,\;i=1,2)$, and environment technology coefficient ($j_{i}>0,\;i=1,2$).

Besides, $G_{1}=G_{1}\left(K_{1}\right)$ indicates the emission rate of pollution at the level of its output. $G_{K,1}>0$, $G_{KK,1}\geq 0$, $G_{1}\left(0\right)=0$ satisfy the first- and second-order conditions and the initial condition. For simplicity, we assumed the pollution is only from the developing country but will affect both countries. An example is the transboundary emission from coal-fired power plants or some waste such as Chlorofluorocarbon (CFC) due to lax environmental standards and regulations in the developing country. These have been banned by the developed country.

Also suppose that $U_{i}\left(C_{i},\;Z\right)$ is a continuous and differentiable function at $C_{i}$ and Z, $U_{C,i}>0$, $U_{Z,i}\leq 0$, $U_{CC,i}<0,\;U_{ZZ,i}\;\leq 0$, and $U_{Z,i}\left(C_{i},0\right)=0$. These mean that marginal utility will increase as consumption increases and decrease as pollution stock increases. That is to say, the marginal utility of consumption is made worse if pollution becomes more severe, and the marginal disutility of pollution is aggravated if more consumption goods were spoiled. Further, suppose the cross effect $U_{CZ,i}=U_{ZC,i}=0$. Although $U_{CZ,i}$ and $U_{ZC,i}$ may be positive or negative, the total marginal utility will not reach the maximum unless they are zero simultaneously when the marginal utility from consumption is quickly offset by the marginal disutility from severe pollution. This is because that $U_{CZ,i}$ and $U_{ZC,i}$ are limited by the economies of scale and unrestricted at their maximum.

Next, we set up the Hamilton function to derive the solution to the dynamic optimization as follows:

(6) $$H=U_{1}\left(C_{1},\;Z\right)+U_{2}\left(C_{2},\;Z\right)+\lambda [F_{1}\left(K_{1}\right)-C_{1}-E_{1}-\delta_{1}K_{1}],$$

where λ indicates the marginal efficiency of capital stock. Based on the first-order conditions $\partial H/\partial C_{1}=0$, $E_{1}\partial H/\partial E_{1}=0$, $E_{2}\partial H/\partial E_{2}=0$, we can have the following:

(7) $$U_{C,1}=\lambda ,$$

(8) $$E_{1}\left[\left(U_{Z,1}+U_{Z,2}\right)\left(-j_{1}\right)-\lambda \right]=0,$$

(9) $$E_{2}\;\left[-\left(U_{Z,1}+U_{Z,2}\right)j_{2}-U_{C,2}\right]=0,$$

Based on the Euler equation $d\left(e^{-\theta t}\lambda \right)/dt=-\partial H/\partial K_{1}$, we have,

(10) $$\dot{\lambda }=\lambda \left(\theta +\delta_{1}-F_{K,1}\right)-\left(U_{Z,1}+U_{Z,2}\right)G_{K,1}.$$

Here, $\dot{\lambda }=d\lambda /dt$ is the derivative of λ with respect to t and measures the rate of change of the shadow value of capital stock. The transversality condition is:

(11) $$\underset{t\to \infty }{\lim}K_{1}\left(t\right)\lambda \left(t\right)e^{-\theta t}=0.$$

## 4. Four Regions of Environmental Investment

In Equations (7) to (11), it is impossible for us to solve these nonlinear and dynamic problems and get their closed-form solutions. This is because there are some compound functions embedded in these equations. To facilitate an effective analysis, we divided the decisions on the environmental investment of both countries into four portfolios: (1) $E_{1}>0$, $E_{2}>0$; (2) $E_{1}>0$, $E_{2}=0$; (3) $E_{1}=0$, $E_{2}>0$; and (4) $E_{1}=E_{2}=0$. Based on the observation of the possible practice to implement the principle of “common but differentiated responsibility”, this is a feasible method to locate the optimal solution on the plane for the space of possible decisions of both countries. Therefore, there are four regions of the possible decision space corresponding to four portfolios for both countries. We will discuss the boundary of four regions on a plane and analyze the optimal approximate solution and evolving path.

### 4.1. Region A

In Region A, both countries invest in environment protection:$\;E_{1}>0$, $E_{2}>0$. From Equation (7), we have $\left(U_{Z,1}+U_{Z,2}\right)j_{1}=-\lambda$. Since the second-order condition of the utility function satisfies $U_{C}=0$, $U_{CZ}=0$, and $U_{ZC}=0$, the partial differentiation of utility on pollution material stock Z, i.e.,$\;U_{Z}$, is decided only by C and Z. Thus, we have $\left[U_{Z,1}\left(C,\;Z\right)+U_{Z,2}\left(C,Z\right)\right]j_{1}$=− λ. The increase of the marginal efficiency of capital can increase the total output, which will subsequently increase the pollution stock Z:

(12) $$Z_{\;\lambda }=\frac{\partial Z}{\partial \lambda }=-\frac{1}{j_{1}\left(U_{ZZ,1}+U_{ZZ,2}\right)}=0.$$

Hence, the pollution stock function has the form Z=Z(λ), which is a monotonically increasing function of the marginal efficiency of capital λ. Since $U_{C,1}=\lambda$ in Equation (7), we can derive the monotonically decreasing function of *λ* for the consumption of the developing country $\;C_{1}=C_{1}\left(\lambda \right)$:

(13) $$C_{\lambda }^{1}=\frac{\partial C_{1}}{\partial \lambda }=\frac{1}{U_{ZZ,1}}<0.$$

From Equations (9) and (10), we can have $\;\lambda =\left(j_{1}/j_{2}\right)U_{C,2}$. Taking the partial differential on it, we have the monotonically decreasing function of *λ* for the consumption of developed country $C_{2}=C_{2}\left(\lambda \right)$:

(14) $$C_{\lambda ,2}=\frac{\partial C_{2}}{\partial \lambda }=\frac{j_{2}}{j_{1}U_{CC,2}}<0.$$

In the developed economy, the total expenditure of variable consumption and environmental investment is $Y_{2}=C_{2}\left(\;\lambda \right)+E_{2}$. Taking the partial differentiation for *λ* and $Y_{2}$, we can find that the environmental investment $E_{2}=E_{2}\left(\;\lambda ,\;Y_{2}\right)$ is a monotonically increasing function of λ and $Y_{2}$. Therefore, the environmental investment of the developed country is decided by the marginal efficiency of capital stock λ and the level of disposable income $Y_{2}$:

(15) $$E_{\lambda ,2}=\frac{\partial E_{2}}{\partial \lambda }=-C_{\lambda ,2}>0,\;\;\;\frac{\partial E_{2}}{\partial Y_{2}}=1>0.$$

The intensity of environment pollution is *Z*($\lambda )=G_{1}\left(K_{1}\right)-j_{1}\left(K_{1}\right)-j_{2}E_{2}\left(\lambda ,\;Y_{2}\right)$, which can be transformed into $E_{1}=1/j_{1}G_{1}\left(K_{1}\right)-Z\left(\lambda \right)-j_{2}E_{2}\left(\lambda ,\;Y_{2}\right)$. Taking the partial differentiation for λ, $K_{1}$, and $Y_{2}$, we have the environmental investment of the developing country decided by marginal efficiency of capital stock, capital stock in developing country, and the disposable income in the developed country. That is, $E_{1}=E_{1}\left(\lambda ,E_{1},Y_{2}\right)$. Using Equations (12) and (15), we derive that the environmental investment function in the developing country is a monotonically decreasing function of λ and $Y_{2}$, but a monotonically increasing function of $K_{1}$. Thus, as the marginal efficiency of capital and the disposable income in the developed country increase, the environmental investment of the developing country will decrease. If the domestic capital stock increases, the environmental investment of the developing country would also increase. That is,

(16) $$\frac{\partial E_{1}}{\partial \lambda }=-\frac{1}{j_{1}}\left(-Z_{\lambda }-j_{2}E_{\lambda ,2}\right)<0,\;\;\frac{\partial E_{1}}{\partial K_{1}}=\frac{1}{j_{1}}G_{K,1}>0,\;\;\frac{\partial E_{1}}{\partial Y_{2}}=-\frac{j_{2}}{j_{1}}\frac{\partial E_{2}}{\partial Y_{2}}<0.$$

According to Equations (5), (7), (8) and (10), we can derive the locus of $\lambda^{*}$ as:

(17) $$\lambda^{*}=-(U_{ZZ,1}+U_{ZZ,2})j_{1}Z.$$

Therefore, we have the following result.

**Proposition 1:**  *When* $E_{1}>0$ *,* $E_{2}>0$ *, the environment pollution intensity moves in tandem with the shadow price of the capital stock in the developing country.*

The proofs see the derivation as following in Equations (19)–(22).

### 4.2. Region B

In Region B, $E_{1}>0$, $E_{2}=0$, and the environmental investment of the developed country is zero, and the developing country has made efforts in governing environment pollution. From Equation (8), the marginal utility of consumption in the developed country is greater than the marginal utility of decreasing pollution in both countries from the environmental protection expenditure in the developing country. That is, −($U_{Z,1}+U_{Z,2}{|}_{E_{2}=0}j_{2}<U_{C,2}$). Equation (12) still holds as $E_{1}>0$. Additionally, pollution stock is $Z\left(\lambda \right)=G_{1}\left(K_{1}\right)-j_{1}E_{1}$, which can be transformed into $E_{1}=1/j_{1}\cdot [G_{1}\left(K_{1}\right)-Z\left(\lambda \right)]$. Taking the partial differentiation on λ and $K_{1}$, we have the environmental investment of the developing country determined by the marginal efficiency of capital stock and the capital stock in the developing country. Using Equations (13) and (14), we can find that the environmental investment function in the developing country is a monotonically decreasing function of λ, but a monotonically increasing function of $K_{1}$. Thus, as the marginal efficiency of capital increase, the environmental investment of the developing country will decrease. If the domestic capital stock increases, the environmental investment of the developing country would also increase:

(18) $$\frac{\partial E_{1}}{\partial \lambda }=-\frac{1}{j_{1}}Z_{\lambda }>0,\;\frac{\partial E_{1}}{\partial K_{1}}=\frac{1}{j_{1}}G_{K,1}<0.$$

Without environmental aids from the developed country, the developing country should increase the environmental investment along with the capital accumulation and economic development. Equation (17) still holds, and $\dot{\lambda }=-\left(U_{ZZ,1}+U_{ZZ,2}\right)j_{1}Z$. The fluctuation locus of the economy system’s environment pollution intensity is the same as the track of the capital stock shadow price of the developing country. Hence, we have the following proposition.

**Proposition 2:**  *Let* $E_{1}>0$ *,* $E_{2}=0$ *, the environment pollution stock moving in tandem with the shadow price of the capital stock in the developing country.*

The proofs see the derivation as following in Equations (19)–(22).

**Figure 1:**
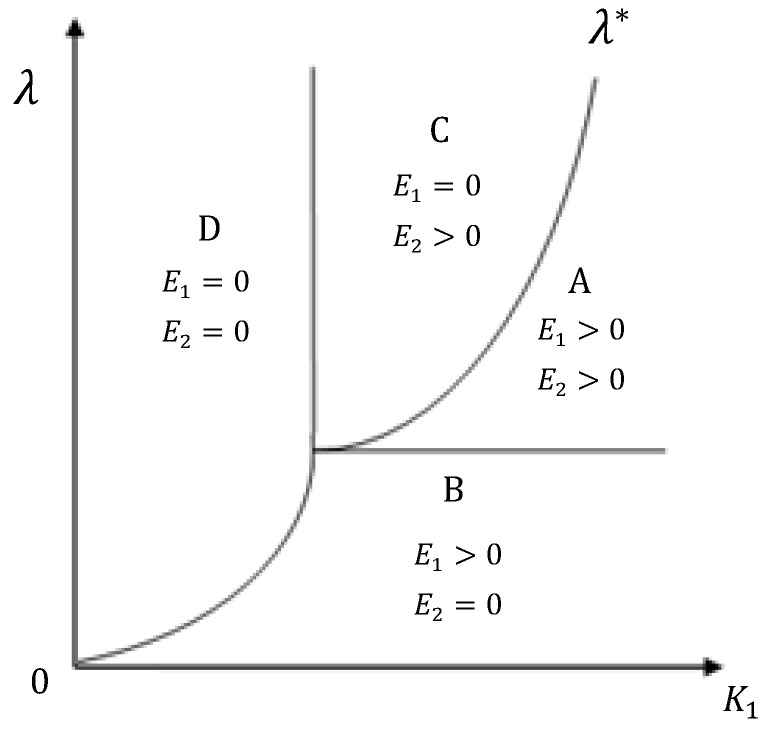
Four regions of environmental investment in the developing and developed countries.

If $E_{1}>0$, Equations (5) and (6) hold, and the pollution stock function has the form: $Z=G_{1}\left(K_{1}\right)-j_{1}E_{1}\left(\lambda ,K_{1},Y_{2}\right)-j_{2}E_{2}\left(\lambda ,Y_{2}\right)$. Hence, Equation (13) holds, and we can have the horizontal boundary between Regions  A and B in the $K_{1}-\lambda$ plane (see Figure 1) as follows:

(19) $$\frac{\partial E_{1}}{\partial \lambda }=\frac{1}{j_{1}}\left(-Z_{\lambda }-j_{2}E_{\lambda ,2}\right)<0,\;\;$$

(20) $$\frac{\partial E_{1}}{\partial K_{1}}=\frac{1}{j_{1}}G_{K}>0,\;\;$$

(21) $$\frac{\partial \lambda }{\partial K_{1}}=\frac{G_{K,1}-j_{1}E_{K,1}}{j_{1}E_{\lambda ,1}+j_{2}E_{\lambda ,2}}=0.$$

Replacing $E_{K,1}$ in (20) with (21), we can see capital return  λ  does not change with capital stock in the developing country, suggesting the boundary is horizontal. The developed country is indifferent on choosing zero or a positive environmental investment on the boundary between Regions A and B. From Equations (5) and (6), the horizontal boundary between Regions A and B is:

(22) $$\lambda =\frac{j_{1}U_{C,2}}{j_{2}}{|}_{C_{2}=Y_{2}}.$$

### 4.3. Region C

In Region C, $\;E_{1}=0$, $E_{2}>0$, and the environmental investment of the developing country is zero, while the developed country makes efforts on pollution control. From Equation (8), the marginal utility of consumption in the developing country is greater than the marginal utility of decreasing pollution in both countries from the environmental investment in the developed country,$-\left(U_{Z,1}+U_{Z,2}\right)\;{|}_{E_{1}=0}j_{1}<U_{C,1}$. From Equation (7), we have $-\left(U_{Z,1}+U_{Z,2}\right)\;j_{1}<\lambda$. According to $U_{CZ,1}=0$ and $\lambda =U_{C,1}\left(C_{1}\right)$ in Equation (7), Equation (13) still holds. Moreover, from Equation (9), we have $-(U_{Z,1}+U_{Z,2})\;j_{2}=U_{C,2}$. The marginal utility of consumption in the developed country is equal to the marginal utility of decreasing pollution in both countries from the environmental protection expenditure in the developing country. Because $U_{Z}$ and $U_{C}$ are both determined by Z and C and $-(U_{Z,1}\left(C_{1},\;Z\right)+U_{Z,2}\left(C_{2},\;Z\right)]j_{2}=U_{C,2}\left(C_{2},\;Z\right)$, we can take the partial differential on $C_{2}$ and Z. The consumption in the developed country will decrease as the pollution stock increases, as the developed country needs to spend more on environmental protection. We have:

(23) $$\frac{\partial C_{2}}{\partial Z}=-\frac{j_{2}(U_{ZZ,1}+U_{ZZ,2})}{U_{CC,2}}<0.$$

When the environmental investment of the developing country is zero, the pollution stock is $Z=G_{1}\left(K_{1}\right)-j_{2}E_{2}$. Using $Y_{2}=C_{2}+E_{2}$, the pollution function can be transformed into $\;Z=G_{1}\left(K_{1}\right)-j_{2}\left(Y_{2}-C_{2}\right)$. The following confirms that Z is an increasing function of $K_{1}$:

(24) $$\frac{dZ}{dK_{1}}=\frac{G_{K,1}}{1-j_{2}C_{Z,2}}>0.$$

Consumption in the developed country can be presented as a function of Z and $K_{1}$ That is, $\;C_{2}=C_{2}\left(Z\left(K_{1}\right)\right)$. Using Equations (23) and (24), we can conclude that the consumption in the developed country would decrease if the capital accumulates faster in the developing country:

(25) $$\frac{dC_{2}}{dK_{1}}=\frac{dC_{2}}{dZ}\times \frac{dZ}{dK_{1}}=\frac{j_{2}(U_{ZZ,1}+U_{ZZ,2})}{U_{CC,2}}\times \frac{G_{K,1}}{1-j_{2}C_{Z,2}}<0.$$

Consequently, the environmental investment in the developed country is $E_{2}=E_{2}\left(K_{1},Y_{2}\right)=Y_{2}-C_{2}(Z\left(K_{1}\right)$. Taking the partial derivative for $K_{1}$ and $Y_{2}$ in the environmental investment function, we have:

(26) $$\frac{\partial E_{2}}{\partial K_{1}}=-\frac{\partial C_{2}}{\partial K_{1}}>0,\;\;\;\frac{\partial E_{2}}{\partial Y_{2}}>0.$$

Hence, we have the following proposition.

**Proposition 3:**  *Let* $E_{1}=0$ *,* $E_{2}>0$ *. The environmental investment in the developed country increases as the capital stock and the disposable income increase in the developing country.*

The proofs see the derivation as follows and Equations (27) and (28).

In Regions A and C, $E_{2}>0$. If $E_{1}=0$, in Region C, from Equations (4) and (6) we can derive Equations (16)–(18). The environmental investment in the developed country ($E_{2}$) is a monotonically increasing function of the capital stock in the developing country. That is,

(27) $$E_{K,2}=\frac{C_{Z,2}G_{K,1}}{1-j_{2}C_{Z,2}}>0.$$

In the boundary between Regions A and C, the developing country is indifferent on choosing zero or a positive environmental investment: $\;E_{1}\geq 0$. Equation (5) indicates $-\left(U_{Z,1}+U_{Z,2}{|}_{E_{1}=0}\right)\;j_{1}=\lambda$. Using the pollution stock function $Z=G_{1}\left(K_{1}\right)-j_{1}E_{2}\left(K_{1}\right)$, we can derive the partial derivative of λ in the boundary:

(28) $$\frac{\partial \lambda }{\partial K_{1}}=-j_{1}\left(U_{ZZ,1}+U_{ZZ,2}\right)\left(G_{K,1}-j_{2}E_{K,2}\right)>0.$$

We can now replace $E_{K,2}$ in Equation (24) with Equation (23). Using Equation (16), we can have $G_{K,1}-j_{2}E_{K,1}=G_{K,1}+j_{2}G_{Z,2}G_{K,1}/\left(1-j_{2}C_{Z,2}\right)=G_{K,1}/\left(1-j_{2}C_{Z,2}\right)$. Hence, the boundary between Regions A and C is a curve with a positive slope which can be derived from.

### 4.4. Region D

In Region D, the marginal utility of consumption in each country is greater than its marginal utility of pollution reduction. Hence, the environmental investment is zero in both countries: $\;E_{1}=E_{2}=0$. From Equations (8) and (9), we can have $-\left(U_{Z,1}+U_{Z,2}\right)\;{|}_{E_{1}=0}j_{1}<\lambda =U_{C,1}\left(C_{1},\;Z\right)$ and $-(U_{Z,1}+U_{Z,2}{|}_{E_{2}=0})<U_{C,2}$. Equations (7) and (13) still hold. Also, all disposable income in the developed country is used for consumption: $Y_{2}=C_{2}$. We have:

(29) $$\dot{Z}=G_{K,1}{\dot{K}}_{1}.$$

Hence, we have the following proposition.

**Proposition 4:**  *Let* $E_{1}=0$ *,* $E_{2}=0$ *, there is no environmental investment in both countries. The pollution stock moves in the same way as the shadow price of the capital stock in the developing country.*

The proofs see the derivation as following:

In the same as the proofs of boundary between Regions A and C in Equations (27) and (28), and (22), we can derive the boundary between Regions B and D as $-\left(U_{Z,1}+U_{Z,2})\;{|}_{E_{1}=E_{2}=0}\right)\;j_{1}=\lambda$. The boundary between Regions C and D is a vertical line derived from:

(30) $$-\left(U_{Z,1}+U_{Z,2}{|}_{E_{1}=E_{2}=0}\right)=U_{C,2}/j_{2}{|}_{C_{2}=Y_{2}}.$$

To summarize, the results on four regions and their boundary are shown on the plane $K_{1}-\lambda$ in Figure 1. All boundaries are determined by the Equations in (7) to (11) and summarized in next section.

### 4.5. Boundaries of the Four Regions

In each of the four regions presented above, the pollution stock and the shadow price of the capital stock in the developing country change in the same direction. This indicates that the economic growth is an important factor for pollution control. The economic growth as well as the environmental deterioration in the developing country might be two sides of the same coin. With the increasing environmental investment, an optimal environmental protection path could be achieved.

We now derive the boundary among the four regions on the $K_{1}-\lambda$ plane:

(1) $E_{2}>0$ is corresponding to Regions A and C, with the boundary:$-\left(U_{Z,1}+U_{Z,2}\right)\;{|}_{E_{1}=0}j_{1}=\lambda$;

(2) $E_{2}=0$ is corresponding to Regions B and D, with the boundary: $-\left(U_{Z,1}+U_{Z,2}\right)\;{|}_{E_{1=}E_{2}=0}j_{1}=\lambda$;

(3) $E_{1}>0$ is corresponding to Regions A and B, with the boundary:$\;\lambda =\left(j_{1}U_{C,2}/j_{2}\right){|}_{C_{2}=Y_{2}}$;

(4) $E_{1}=0$ is corresponding to Regions C and D, with the boundary:$-\left(U_{Z,1}+U_{Z,2}\right)\;{|}_{E_{1}=E_{2}=0})=(U_{C,2}j_{1}/j_{2}){|}_{C_{2}=Y_{2}}$;

Hence, we have the following proposition.

**Proposition 5:**  *All four boundary intersect at the same point* $\left(\bar{K},U_{C,2}j_{1}/j_{2}\right)$ *.*

The proofs on Proposition 5 see the derivation as following in Equations (31) to (35).

The intersection point of the common border line between Regions B and D and that between Regions C and D can be derived based on the following simultaneous equations:

(31) $$-\left(U_{Z,1}+U_{Z,2}{|}_{E_{1}=E_{2}=0}\right)j_{1}=\lambda$$

(32) $$-\left(U_{Z,1}+U_{Z,2}{|}_{E_{1}=E_{2}=0}\right)=\frac{U_{C,2}}{j_{2}}{|}_{E_{1}=E_{2}=0}$$

(33) $$Z=G_{1}\left(K_{1}\right)$$

Equation (31) can be transformed into $-\left(U_{Z,1}+U_{Z,2}{|}_{E_{1}=E_{2}=0}\right)=\lambda /j_{1}$. Replacing the left-hand side of Equation (32), we get $\lambda =U_{C,2}j_{1}/j_{2}$. Let the capital stock which equalizes (32) and (33) be a constant $\bar{K}$, we have the intersection point $\left(\bar{K},\;U_{C,2}j_{1}/j_{2}\right)$.

The intersection point of the common border line between Regions A and B and that between Regions B and D can be derived based on the following simultaneous equations:

(34) $$\lambda =\frac{j_{1}U_{C,2}}{j_{2}}{|}_{C_{2}=Y_{2}}$$

(35) $$-\left(U_{Z,1}+U_{Z,2}{|}_{E_{1}=E_{2}=0}\right)j_{1}=\lambda$$

The point of intersection is still $\left(\bar{K},\;U_{C,2}j_{1}/j_{2}\right)$. In the same vein, the intersection point of the common border line between Regions A and C and that between Regions A and B can be obtained in the same way.

## 5. An Optimal Pollution Control Model

In this section, we explore the optimal pollution control and economic growth path. Obviously, general functional forms of utility, pollution, and production functions are intractable to obtain the analytic equilibrium of the pollution control model. In order to clear the market and analytically determine the optimal economic growth path, the related functional forms of utility, pollution, and production are set as follows [37]:

(36) $$U=\frac{C^{1-\sigma }-1}{1-\sigma }-\frac{Z^{1+\rho }}{1+\rho },\;0<\sigma \leq 1,\;\;\rho >0,$$

(37) $$Z=K^{\beta }-j_{1}E_{1}-j_{2}E_{2},\;\;j>0,\;\;\beta \geq 1,$$

(38) $$F=bK^{\alpha },\;b>0,\;\;0<\alpha <1.$$

Here, σ is the elasticity coefficient of marginal utility from consumption, ρ denotes the elasticity coefficient of marginal negative utility from pollution, b is a coefficient on capital stocked volume, α is the elasticity coefficient of marginal production. Therefore, we can derive some conclusions as follows.

**Proposition 6:**  *(1)* $\;U_{C}=C^{-\sigma }>0$ *,* $U_{CC}=-\sigma C^{-\sigma -1}<0$ *; (2)* $U_{Z}=-Z^{\rho }<0$ *,* $U_{ZZ}=-\rho Z^{\rho -1},\;U_{ZC}=0,<0$ *; (3)* $Z_{K}=\beta K^{\beta -1}$ *,* $Z_{KK}=\beta \left(1-\beta \right)K^{\beta -2}>0$ *; (4)* $Z_{E}=-j<0$ *; (5)* $F_{K}=abK^{\alpha -1}>0$ *,* $F_{KK}=ab\left(\alpha -1\right)K^{\alpha -2}<0$ *; (6)* $U_{CC}C/U_{C}=-\sigma <0$ *; (7)* $\;U_{ZZ}Z/U_{Z}=\rho >0$ *;* *(8)* $F_{KK}K/F_{K}=\alpha -1<0$ *.*

The proofs on Proposition 6 see the derivation as following in Equations (39) to (48).

In Region A, $E_{1}>0$, $\;E_{2}>0$. From Equations (5) and (7), we have $\dot{\lambda }=-\left(U_{ZZ,1}+U_{ZZ,2}\right)\left[j_{1}\left(\theta +\delta_{1}-F_{K,1}\right)-G_{K,1}\right]$. At the steady state of the economic growth path of the developing country, the capital return should be stable. So, $\dot{\lambda }=0$ and $U_{Z}<0$ will give $j_{1}\left(\theta +\delta_{1}-F_{K,1}\right)-G_{K,1}=0$. Hence, the economic growth path of the developing country should satisfy the following:

(39) $$\theta +\delta_{1}+\frac{G_{K,1}}{j_{1}}-F_{K,1}=0.$$

Using Equations (26) and (27), we can calculate $G_{K,1}=\beta K^{\beta -1}$ and $F_{K,1}=b\alpha K^{\alpha -1}$. Equation (39) can be transformed into:

(40) $$\theta +\delta_{1}+\beta \frac{K^{\beta -1}}{j_{1}}-\alpha K^{\alpha -1}=0.$$

We can further calculate the capital stock in the equation by assuming β=1, and we can obtain the optimal capital stock of the developing country at the steady state as:

(41) $$K_{1}=\tilde{K}={\left(\frac{\theta +\delta_{1}+\frac{1}{j_{1}}}{b\alpha }\right)}^{\frac{1}{\alpha -1}}.$$

Since $U_{C}=C^{-\sigma }$, $U_{Z}=Z^{\rho }$ and $U_{C}^{1}=\lambda$ in Equation (4), we can get the optimal consumption in the developing country as:

(42) $$C_{1}=\lambda^{-\frac{1}{\sigma }}.$$

From Equations (5) and (6), $U_{C,2}={\left(C_{2}\right)}^{-\sigma }j_{2}/j_{1}$, and we have the optimal consumption in the developed country as:

(43) $$C_{2}={\left(\frac{j_{2}\lambda }{j_{1}}\right)}^{-\frac{1}{\sigma }}.$$

From Equation (43) and $U_{Z}=-Z^{\rho }-j_{1}\left(U_{Z,1}+U_{Z,2}\right)=\lambda$, using $G_{1}\left(K_{1}\right)=K_{1}$, $Y_{2}$= $C_{2}$ + $E_{2}$, and Equation (43) and Equation (13), we can derive the optimal pollution stock as follows:

(44) $$E_{1}=\frac{1}{j_{1}}\left\{K_{1}-{\left(\frac{\lambda }{2j_{1}}\right)}^{\frac{1}{\rho }}-j_{2}Y_{2}+j_{2}{\left(\frac{j_{2}\lambda }{j_{1}}\right)}^{-\frac{1}{\sigma }}\right\}.$$

**Figure 2:**
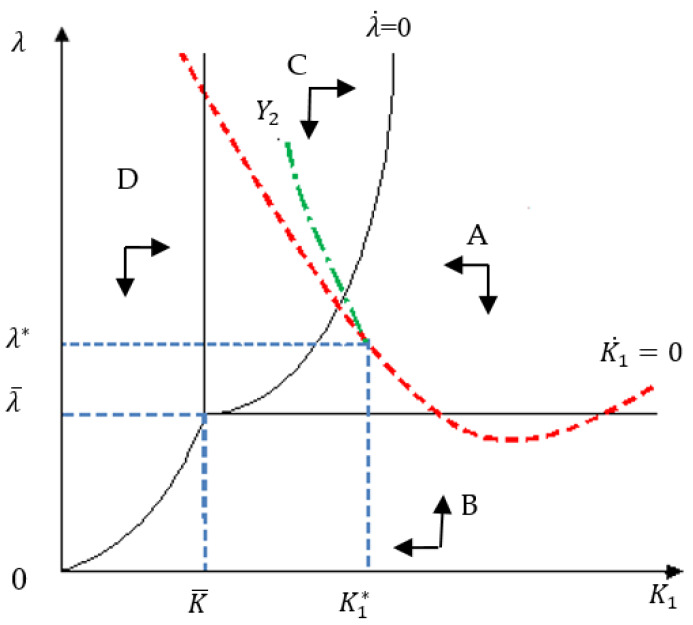
The phase diagram on the optimal economic growth path for the developing country.

The steady state of the capital accumulation in the developing country suggests ${\dot{K}}_{1}=0$. Hence, the capital accumulation condition in Equation (1) is ${\dot{K}}_{1}=F_{1}\left(K_{1}\right)-C_{1}\left(\lambda \right)-E_{1}\left(K_{1},\;\lambda \;\right)-\delta_{1}K_{1}=0$. Using Equations (29), (42), and (45), the optimal condition of the capital accumulation in the developing country as well the optimal economic growth path is given in Figure 2:

(45) $$b{\left(K_{1}\right)}^{\alpha }-{\left(\lambda \right)}^{-\frac{1}{\sigma }}-\frac{1}{j_{1}}\left\{K_{1}-{\left(\frac{\lambda }{2j_{1}}\right)}^{\frac{1}{\rho }}-j_{2}Y_{2}j_{2}+{\left(\frac{j_{2}\lambda }{j_{1}}\right)}^{-\frac{1}{\sigma }}\right\}-\delta_{1}K_{1}=0.$$

Equations (42) and (45) jointly determine the equilibrium point $\left(\tilde{K},\;\lambda^{*}\right)$. Next, we analyze the boundary between Regions C and D. When the developing country provides no expenditure on environmental protection, i.e., $\;E_{1}=0$, the boundary between Regions C and D gives the contingent condition that the developed country is indifferent on whether to spend on environmental protection, $E_{2}>0$, or not, $E_{2}=0$. Hence, given $E_{1}=0$ and $E_{2}=0$, we have $\;Z=Z=G_{1}\left(K_{1}\right)=K_{1}$ and $C_{2}=Y_{2}$. The boundary can be rewritten as $-j_{2}\left[U_{Z,1}\left(G_{1}\left(K_{1}\right)\right)+U_{Z,2}\left(G_{1}\left(K_{1}\right)\right)\right]=U_{C,2}\left(Y_{2}\right)$. Using $U_{C}=C^{-\sigma }$ and $U_{Z}=-Z^{\rho }$, the boundary is $2j_{2}{\left(K_{1}\right)}^{\rho }={\left(Y_{2}\right)}^{-\sigma }$. We can calculate the boundary as:

(46) $$K_{1}=\bar{K}={\left(\frac{{\left(Y_{2}\right)}^{-\sigma }}{2j_{2}}\right)}^{\frac{1}{\rho }}.$$

Comparing (41) and (46), we can derive that the necessary condition of the equilibrium in Region A is $\;\tilde{K}>\bar{K}$. That is,

(47) $${\left(\frac{\theta +\delta_{1}+\frac{1}{j_{1}}}{b\alpha }\right)}^{\frac{1}{\alpha -1}}>{\left(\frac{{\left(Y_{2}\right)}^{-\sigma }}{2j_{2}}\right)}^{\frac{1}{\rho }}.$$

We further simply the inequality by assuming σ=1,  ρ=1,  α=1/2, and obtain the necessary condition of equilibrium being within Region A as:

(48) $$Y_{2}>\frac{2{\left(+\delta_{1}+\frac{1}{j_{1}}\right)}^{2}}{b^{2}j_{2}}.$$

Therefore, as the disposable income of the developed country is more than the value in Equation (48), the developing country can accumulate the capital stock beyond Region D and afford the cost of environmental protection.

### 5.1. The Optimal Equilibrium

The equilibrium of the pollution control model should distribute the environmental protection cost between the two countries. Hence, the optimal economic growth path of the developing country needs to converge to a point within Region A.

As detailed in the proofs on Proposition 6 in Equations (39) to (48), if the disposable income of the developed country is more than the value in Equation (49), we have the following proposition.

**Proposition 7:**  *The developing and developed countries may obtain maximum social welfare at a stable equilibrium point* $\left(K_{1}^{*},\;\lambda^{*}\right)$ *, which would be within Region A if and only if environmental investment* $Y_{2}$ *satisfies the inequality equation as follows:*

(49) (θ+δ1+1j11−σ)−2>(Y2)−12j2,    Y2>2(θ+δ1+1j1)2b2j2.

The proofs for the Proposition 6 is the same as the derivation in Equations (39) to (48) for the Proposition 6. Therefore, the four regions, their boundary, the intersect point, and optimal equilibrium point are shown as the plane $K_{1}-\lambda$ in Figure 2.

In Figure 2, four boundaries intersect at the point $\left(\bar{K},\;\bar{\lambda }\right)$. Here, $\bar{K}={\left({\left(Y_{2}\right)}^{-\sigma }/2j_{2}\right)}^{\frac{1}{\rho }}$, $\bar{\lambda }=U_{C,2}j_{1}/j_{2}$. Under the principle of “common but differentiated responsibility” in the Paris Agreement, the developing and developed countries may cooperate with sharing advanced environmental technologies and increasing financial support and reach an approximate solution at the point $\left(K_{1}^{*},\;\lambda^{*}\right)$ on the curve of $\dot{K_{1}}=0$. This means that both countries will reach a stable dynamic equilibrium with keeping a marginal efficiency of capital stock at $\lambda^{*}$ and the rate of increase of shadow value of this capital stock (economic growth) at $\dot{\lambda }=d\lambda /dt$

### 5.2. Discusion and Insights

This condition equilibrium shows that the developing country is willing to control its environment pollution issues if and only if the disposal income from the developed country is more than $2{\left(\delta +\theta_{1}+1/j_{1}\right)}^{2}/b^{2}j_{2}$. This means that the developed country should invest in its own environment technology and help improve the environment technology in the developing country at the initial stage of economic growth. This is because that the economic growth and technological improvement could help the developing country to improve the capital return λ as well as the capital formation $K_{1}$ into Region A. This result is consistent with the environmental Kuznets curve (EKC) hypothesis [34]. However, as the disposable income of the developed country is more than the value in Equation (49), the capital stock of the developing country can be accumulated beyond Region D and afford the environmental protection cost at a late stage of economic growth in emerging countries such as China, Brazil, and Saudi Arabia. Along the optimal trajectory path $\dot{K_{1}}=0$, the economic growth increases quickly at the initial stage, then decreases when entering Region A, and ultimately increases with an increase in the environmental investment in the developing country. This means that global economic growth will decrease even though the developed country takes more responsibilities for the environmental protection costs and increase if and only if the developing country takes more responsibility for environmental protection costs at the expense of economic growth at the late stage and call the developed country back to invest in environmental protection. Maybe, this is the reason why the U.S. withdrew from the Paris Agreement. This result is different with the EKC hypothesis at a late stage of economic growth in the developing country.

Moreover, according to the accumulation of capital stocked volume $K_{1}$, the optimal economic growth approach of the developing country can be divided into three stages, as illustrated in Figure 2. In the first stage, in Region D, the capital stock level of the developing country is too low to afford any environmental protection, thus $E_{1}=0$. Also, the developed country has insufficient disposal income to invest in pollution control for the developing country ($E_{2}=0$). The consumption in the developing country $C_{1}>0$ increases continuously so that its marginal utility $U_{C,1}$ keeps declining. According to Equation (4), the capital return is equal to the marginal utility of consumption in the developing country. Hence, as the capital stock increases in the developing country, the capital return  λ would decrease along the optimal economic growth curve into Region D (see Figure 2). The consumption of the developed country is at a steady state, but the sum of pollution stock $Z=G_{1}\left(K_{1}\right)$ increases continuously, which deteriorates the environment of both countries.

In the second stage, after the capital stock goes beyond Region D (i.e., $\tilde{K}=\bar{K}$), the optimal economic path crosses into Region C. However, the capital stock of the developing country is still insufficient to afford any cost for pollution control. The environmental investment in the developing country is still $E_{1}=0$, while the developed country now has enough disposable income to invest in pollution control ($E_{2}>0$).

As the capital stock accumulates in the developing country, the economy grows and the consumption ($C_{1}$) keeps increasing. The marginal utility of consumption ($U_{C,2}$) and the capital return (λ) decline along the optimal economic growth curve ($E_{1}=0$) in Region C (see Figure 2). On the one hand, according to Equation (23), the environmental investment in the developed country ($E_{2}$) increases with its increasing disposable income ($Y_{2}$) and capital stock accumulation in the developing country ($K_{1}$). Along with the increasing consumption ($C_{1}$) in the developing country, the sum of pollution stock Z also increases continuously. Therefore, the developed country needs to moderately decrease its domestic consumption ($C_{2}$) to increase environmental investment ($E_{2}$), as follows:

(50) $$C_{1}↑\to \lambda ↓\;\to K_{1}↑\;\left(\begin{array}{c} \frac{\partial E_{2}}{\partial E_{1}}>0\\ \frac{\partial Z}{\partial K_{1}}>0 \end{array}\right)\to \;\{\begin{array}{c} E_{2}↑\to \;\;C_{2}↓\\ Z↑\;\;\;\;\;\;\;\;\;\;\;\;\;\;\;\; \end{array}.$$

In the third stage, in Region A ($E_{1}>0,$ $E_{2}>0$), when the capital stock level of the developing country is high enough to invest in environmental protection, the environmental investment from both countries increases continuously to reduce pollution. As the capital stock keeps increasing, the capital return (λ) decreases in the developing country. According to Equation (13), the consumption in the developing country ($C_{1}$) increases continuously. At the same time, Equations (15) and (16) indicate increasing environmental investment in the developing country ($E_{1}$) but decreasing environmental investment in the developed country ($E_{2}$). According to Equation (14), the consumption in the developed country ($C_{2}$) can resume step by step, as this can save environmental investment. According to Equation (12), the pollution stock (C) begins to decrease in this stage.

(51) $$C_{1}↑\to \lambda ↓\;\to \;\begin{array}{c} \frac{\partial C_{2}}{\partial \lambda }<0,\frac{\partial E_{1}}{\partial \lambda }<0\;\\ \frac{\partial E_{2}}{\partial \lambda }>0,\frac{\partial Z}{\partial \lambda }>0 \end{array}\}\to \;C_{2}↑,\;\to E_{1}↑,E_{2}↓,\;Z↓\;\;$$

This shows that the total stock of pollution *Z* in both countries will decrease. The consumption will recover if the developing (developed) country continuously increases (decreases) its environmental investment.

Based on the analysis above, we can explore the effect of increasing environmental investment of the developed country. In Regions C and D, the developing country has no environmental investment ($E_{1}=0$). The consumption in the developed country ($C_{2}$) increases along with its disposable income ($Y_{2}$). The marginal utility of consumption in the developed country ($U_{C,2}$) keeps decreasing. Hence, the boundary $-\left(U_{Z,1}+U_{Z,2}\right)\;{|}_{E_{1}=E_{2}=0})=(U_{C,2}j_{1}/j_{2}){|}_{C_{2}=Y_{2}}$ of Regions C and D would move leftwards (See the derivation in Equation (39) to (48) for the Proposition 6). As the disposable income ($Y_{2}$) increases in the developed country, the common border line between Regions C and D, i.e., K1=K¯=(Y2)−σ/2j2)Iρ, would also move leftwards). In Regions A and B, the developing country invests in environmental protection $\;E_{1}>0$. The consumption in the developed country ($C_{2}$) increases along with its disposable income ($Y_{2}$). As the marginal utility of consumption in the developed country ($U_{C,2}$) and capital return (λ) decrease, the boundary $\lambda =(U_{C,2}j_{1}/j_{2}){|}_{C_{2}=Y_{2}}$ of Regions A and B shifts downwards.

In Regions A and C, the developed country invests in environmental protection ($E_{2}>0$). The boundary between Regions A and C is $-\left(U_{Z,1}+U_{Z,2}{|}_{E_{1}=0}\right)j_{1}=\lambda$. In Region C, $E_{1}=0$ and $Z=G_{1}\left(K_{1}\right)-E_{2}j_{2}$. Using Equation (19), we can see that as $Y_{2}$ increases, the pollution stock Z decreases. That is,

(52) $$\frac{\partial Z}{\partial Y_{2}}=\frac{\partial Z}{\partial E_{2}}\frac{\partial E_{2}}{\partial Y_{2}}=-E_{Y,2}j_{2}=-j_{2}<0.\;$$

**Figure 3:**
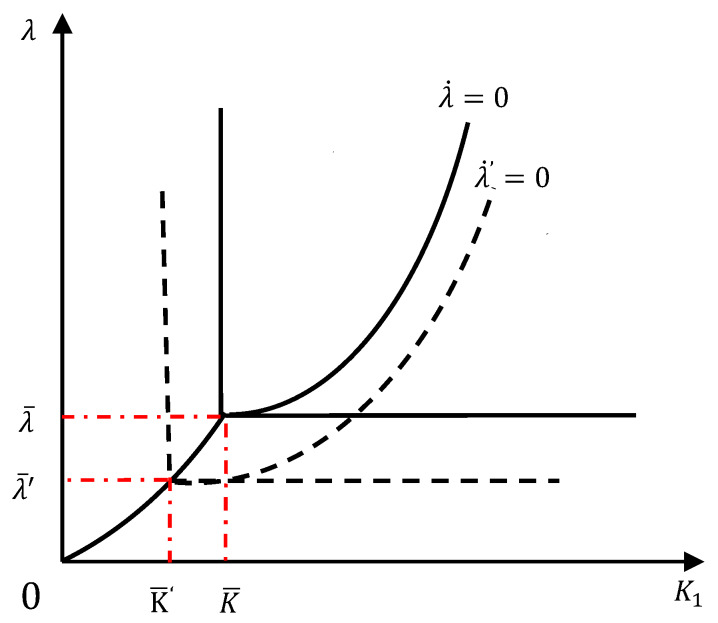
Effects of environmental investment on the developed country.

At the same time, a decreasing  Z decreases $-U_{Z,1}$ and $-U_{Z,2}$. The capital return (λ) decreases with the increasing capital stock in the developing country. Therefore, the boundary between Regions A and C moves downwards and rightwards. In Regions B and D, the developed country does not invest in environmental protection ($E_{2}=0$). The boundary between these two regions is $-\left(U_{Z,1}+U_{Z,2}{|}_{E_{1}=E_{2}=0}\right)\;j_{1}=\lambda$. In Region D, $E_{1}$ = 0, so the pollution stock is only dependent on the pollution emission in the developing country. That is, $Z=G_{1}\left(K_{1}\right)$. In this case, even if the disposable income of developed country ($Y_{2}$) increases, the pollution stock Z does not change with $Y_{2}$. Thus, the boundary between Regions B and D is stationary. These results are depicted in Figure 3.

Intuitively, it is more difficult for the developing country to reduce consumption and capital accumulation at the initial stage of economic growth to protect environment. This is because the main objective of the developing country is to prioritize getting rid of poverty at the cost of environmental protection at this stage. It tends to invest less capital in environmental protection (located in and Regions C and D) to increase its consumption and capital accumulation. This will also increase the stock of pollution in the developing country and result in transboundary pollution and international trade to endanger the developed country. On the other hand, if the developed country chooses to invest in Regions B and D, the stock of pollution will accumulate in the developed country. As a result, this will lead to a worldwide contest in pollution emission between both countries and further deteriorate the global environment. To improve this condition and implement the “common but differentiated responsibility” under the Paris Agreement, it is necessary for both countries to move their investment decision set to Regions A to reach a stable equilibrium mechanism to maximize the social welfare. To do this, it is necessary for the developed countries to enhance cooperation with the developing ones through sharing advanced environmental technologies and increasing financial support at the initial stage of economic growth of the developing country and simultaneously moderately control its domestic consumption.

At a later stage, as the developing country can afford to share the cost of pollution control and achieved advanced environmental technology, the developed country can smooth out the consumption to a normal level with an improved environment. This activity will move the boundary down to the position, as shown in the dotted line from $\left(\bar{K},\;\bar{\lambda }\right)$ to $\left({\bar{K}}^{\prime },{\bar{\lambda }}^{\prime }\right)$ in Figure 3. In such new equilibrium, an optimal pollution control and economic growth path will be reached for developing and developed countries, both of which face the same dual targets of environmental protection and economic growth. In summary, our research shows that there is an optimal growth path for both developed and developing countries to achieve the dual objectives of economic growth and environmental protection in the long run.

## 6. Conclusions

To help achieve the dual goals of pollution control and economic growth around the world, we developed a pollution control model based on a dynamic differential game where a stable equilibrium polices can be reached for cooperation between developing and developed countries. Our results can help with the negotiation on how the agreement would be implemented transparently and fairly for all. Our results show that there is an optimal economic growth for the developing country as well pollution control for both countries. Hence, our results provide a solid theoretical justification for the developing country to share the “common but differentiated responsibilities” with the developed countries, as emphasized in the Kyoto Protocol and the Paris Agreement. Specifically, in the early stage of developing countries’ economic growth and capital accumulation, developed countries need to increase environmental investment to assist the developing countries. As developing countries continue to improve their economy, they can afford to invest in pollution control, which will reduce the required environmental investment from the developed countries. Furthermore, in this way, the developing and developed countries can achieve pollution control without significant loss of social welfare. It is worthwhile noting that as our models can help achieve fair allocation of environmental investment, this can reduce pollution and protect the environment and hence help achieve better health for all, especially the most vulnerable.

There are several limitations in this research. First, in our approach, we limited ourselves to the two variables of pollution and capital. There are various other variables, such as international trade, technological spillovers, and global value chain embedment. Thus, future research can extend our model to a more general setting by incorporating more variables. To identify the critical variables in a general model, principal component analysis can be conducted. Second, empirical studies can be done to test the validity of our models. Third, our model can be also applied to different regions within the same country, which may be in different stages of development. Fourth, in this paper, for simplicity, we assumed the pollution is only from the developing country but will affect both countries. In reality, the developed country also generates some pollution which will affect both countries. Hence, future research can modify our models to incorporate this reality. Fifth, research can be extended to the level of firms, where their strategic interactions and corporate social responsibility strategies can be analyzed [38,39]. Finally, it must be recognized how complex it is to build and simulate a realistic pollution control model. On the other hand, the results obtained in this paper represent a perspective that allows us to establish through numerical modeling that one day we can control environmental pollution so that it leads to the economic development of many nations in the world, which could have multiple repercussions for human health.
